# Role of Th2, Th17 and Treg Cells and relevant cytokines in pathogenesis of allergic rhinitis

**DOI:** 10.1186/s13223-024-00905-8

**Published:** 2024-07-20

**Authors:** Li-Ping Guo, Min Yan, Rui-Bing Niu, Lei Liu, Jing-Ru Yang, Rui-Lian Chen, Bao-Sheng Duan, Cui-Cui Li, Jian-Xiong Li

**Affiliations:** 1 Department of Dermatology, Ordos Central Hospital, Ordos, 017000 Inner Mongolia China; 2 Department of Clinical Laboratory, Ordos Central Hospital, No. 23, Ejinholo West Street, Dongsheng District, Ordos, 017000 Inner Mongolia China; 3 Ordos Clinical Medical College of Inner Mongolia Medical University, Ordos, 017000 Inner Mongolia China; 4 Department of Otolaryngology, Ordos Central Hospital, Ordos, 017000 Inner Mongolia China

**Keywords:** Th2, Th17, Treg, Allergic rhinitis

## Abstract

**Objective:**

To explore the role of different cells and molecules in the pathogenesis of allergic rhinitis (AR) with positive Artemisia allergen by detecting their expression levels.

**Methods:**

From January 2021 to December 2022,200 AR patients diagnosed in the Otolaryngology Clinic of Ordos Central Hospital were selected as the AR group, and 50 healthy people who underwent physical examination in the hospital during the same period were randomly selected as the healthy control (HC) group. The levels of GATA-3mRNA, RORγtmRNA and FoxP3mRNA in peripheral blood mononuclear cells were detected by real-time fluorescence quantitative PCR (qRT-PCR). The proportions of Th2, Th17 and Treg cells were detected by flow cytometry. The concentrations of IL-4, IL-5, IL-17 and IL-10 in serum were detected by enzyme-linked immunosorbent assay. The differences of transcription gene level, immune cell ratio and cytokine concentration between the two groups were analyzed.

**Results:**

There was no difference in age and gender between the two groups. The levels of GATA-3mRNA and RORγtmRNA transcription genes in peripheral blood mononuclear cells, the percentage of Th2, Th17 and Treg immune cells, the levels of eosinophils and basophils in peripheral blood, the concentrations of IL-4, IL-5, IL-17, IL-10 cytokines and IgE in serum of AR patients were significantly higher than those in HC group (*P* < 0.05). IL-4 and IL-17 were positively correlated with total IgE level.

**Conclusion:**

The secretion of immune cells and cytokines in peripheral blood of AR patients is abnormal. Th2, Th17, Treg specific transcription factors and related cells and cytokines are involved in the occurrence and development of allergic rhinitis.

## Introduction

Allergic rhinitis (AR) is an allergic disease characterized by nasal itching, frequent sneezing, a large amount of watery nasal discharge and nasal congestion, and accompanied by symptoms such as itching, redness and swelling of the eyes, and hyposmia in severe cases [1]. It has been demonstrated that the global prevalence of AR is as high as 20%, and the prevalence among Chinese adults has increased from 11.1 to 17.6% within 7 years [2]. In recent years, the number of people suffering from AR in Ordos city has been increasing year by year, presenting a younger-age trend. Artemisia pollen and common inhalational ragweed allergens are the main causes of seasonal AR in Ordos. The positive rate of inhalational allergen Artemisia argyi in autumn is up to 50.7% [3]. Therefore, it is of great significance to establish prevention and treatment strategies targeting local allergens.

There is no breakthrough point in AR treatment. Commonly used antihistamines and corticosteroids can rapidly alleviate the symptoms of AR, but their long-term efficacy is poor with many side effects. The highly effective allergy immunotherapy faces practical difficulties such as long treatment cycles and high costs. Surgical intervention, as a second-line treatment for AR, has certain clinical advantages in relieving stubborn and organic nasal congestion, as well as mucosal hypersecretion and hypersensitivity in AR patients, but may cause postoperative complications such as xerophthalmia, ocular motility disorders, hard palate numbness and toothache [4]. Targeted therapy provides a new direction for personalized treatment of AR, and drug development targeting IgE may become one of the important strategies for AR treatment in the future. The symptoms of AR can be alleviated through medication, but this is only a temporary inhibition of IgE-mediated allergic reactions, and its immunological mechanism needs further in-depth research.

For a long time, Th1/Th2 imbalance has been widely accepted as an allergic disease caused by excessive differentiation of Th2 cells, and Th2 cells play an important role in the pathogenesis of AR [5–7]. According to the research, multiple cytokines, inflammatory cells and inflammatory mediators are jointly involved in the pathogenesis of AR. It is of great significance to further study the mechanism of immune cells and their secreted cytokines in AR.

On this basis, this study detected the expression of Th2/Th17/Treg-related transcription factors and cytokines in the peripheral blood of AR patients, and analyzed the role of these immune cell populations and their functional molecules in the pathogenesis of AR, so as to provide new ways and methods for AR treatment.

## Materials and methods

### Subjects

From January 2021 to December 2022, 200 AR patients diagnosed in the Otolaryngology Clinic of Ordos Central Hospital were selected as the AR group, and 50 healthy people who underwent physical examination in the hospital during the same period were randomly selected as the healthy control (HC) group. The levels of GATA-3mRNA, RORγtmRNA and FoxP3mRNA in peripheral blood mononuclear cells were detected by real-time fluorescence quantitative PCR (qRT-PCR). The proportions of Th2, Th17 and Treg cells were detected by flow cytometry. The concentrations of IL-4, IL-5, IL-17 and IL-10 in serum were detected by enzyme-linked immunosorbent assay.

The inclusion criteria for the healthy control group were as follows: allergen skin prick test-negative and serum allergen-specific IgE-negative, no specific immunotherapy or glucocorticoid therapy before the experiment, and signed informed consent.

The inclusion criteria for the AR group were as follows: All AR patients met the diagnostic criteria in the literature [1], with 2 or more of the symptoms including sneezing, watery discharge, nasal itching and nasal congestion, which lasted or were accumulative for more than 1 h every day, or accompanied by ocular symptoms such as itching and tearing. Additionally, the patients exhibited signs of nasal mucosal edema and watery nasal secretions upon physical examination. The diagnosis of Artemisia allergy was confirmed by experienced clinicians based on a positive allergen inhalation test (AIT) for Artemisia allergens, indicating sensitivity to these specific allergens, as well as positive serum-specific IgE tests against Artemisia allergens. The presence of subjective symptoms as described above, along with positive AIT and IgE tests, established the diagnosis of AR.

The exclusion criteria: Medical history inquiry showed no upper respiratory tract infection within 2 weeks, and excluded bronchiectasis, autoimmune diseases and allergic asthma; patients received specific immunotherapy and glucocorticoid therapy in the past month; patients were complicated with other nasal diseases, or severe lesions of the heart, liver, kidney and other organs; patients were unwilling to participate in this study.

The inclusion criteria for healthy individuals included: allergen skin prick test-negative and serum allergen-specific IgE-negative, no specific immunotherapy or glucocorticoid therapy before the experiment, and signed informed consent.

This study was approved by the Ethics Committee of Ordos Central Hospital (ethical approval No.: 2021-030), and all subjects signed the informed consent.

### Methods

#### Collection of blood samples

Three tubes of fasting peripheral venous blood were aseptically collected from confirmed AR patients and healthy controls in the morning of the second day, including 5 ml blood in an anticoagulant tube containing heparin sodium, 2 ml blood in an EDTA-K2 anticoagulant tube, and 3 ml blood in a separating gel coagulation-promoting tube. After anticoagulant blood collection, repeated reversal was needed 8–10 times for full mixing. All blood samples were processed within 2 h after collection.

#### Detection of Th2, Th17 and Treg cell proportions in peripheral blood

Two Falcon tubes were added with 200 µl heparin sodium anticoagulant whole blood and 4 µl stimulant, respectively, followed by even mixing and incubation in a water bath at 37℃ for 6 h. CD4 FITC fluorescent antibody (20 µl) was added to each tube, which was mixed well and incubated at room temperature in the dark for 15 min. Perm/wash buffer I (100 µl) was added and mixed well for incubation at room temperature in the dark for 5 min. 1X BD FACS lysing solution (2 ml) was added and well mixed, followed by incubation at room temperature in the dark for 10 min, and centrifugation at 800 g for 5 min. After discarding the supernatant, 50 µl perm/wash buffer II was added. One tube was added with 20 µl IL-4 PE fluorescent antibody, and the other with 20 µl IL-17 A PE fluorescent antibody, subjected to mixing well and incubation at room temperature in the dark for 30 min. After washing the cells with PBS, 500 µl PBS was added to each tube and mixed well. The proportions of Th2 and Th17 cells were analyzed using BD FACSCalibur flow cytometry. During the experiment, controls of the same type were set, with CD4^+^IL-4^+^ as control for Th2 cells and CD4^+^IL-17^+^ for Th17 cells.

A Falcon tube was added with 100 µl heparin sodium anticoagulant whole blood, and then added with 20 µl CD25 APC fluorescent antibody and 20 µl CD4 FITC fluorescent antibody, followed by well mixing and incubation at room temperature in the dark for 15 min. 1X BD FACS lysing solution (2 ml) was added and well mixed for red blood cell lysis at room temperature in the dark for 10 min. After washing cells twice with PBS, the supernatant was discarded and the freshly configured FoxP3 Fix/Perm buffer was added. The cells were incubated at room temperature in the dark for 40 min for fixation, and then perm/wash buffer was added to break the cell membrane. After washing the cells with PBS, 20 µl FoxP3 PE fluorescent antibody was added and mixed well, followed by staining at room temperature in the dark for 40 min. After cell washing with PBS, 500 µl PBS was added and mixed well, and the proportion of Treg cells was analyzed by BD FACSCalibur flow cytometer. During the experiment, controls of the same type were set, with CD4^+^CD25^+^FoxP3^+^ as control for Treg cells.

#### Extraction of total RNA from peripheral blood mononuclear cells

Heparin sodium anticoagulant blood (4 ml) was taken and added with 4 ml 0.01 M PBS, which was mixed well and then slowly added into a centrifuge tube containing 4 ml lymphocyte separation medium. After centrifugation at 2,000 rpm/min at room temperature for 15 min, the white cloudy layer dominated by mononuclear cells was sucked into a 10-ml centrifuge tube, which was added with 5 ml 0.01 M PBS and mixed well for centrifugation at 1,500 rpm/min for 10 min and supernatant discarding. After repeated washing twice, mononuclear cells were collected into a 1.5-ml EP tube. Total RNA was extracted from mononuclear cells according to the instructions of the total RNA extraction kit of Tiangen Biotech (Beijing) Co., Ltd. After extraction, the concentration and purity of RNA were determined using an ultraviolet (UV) spectrophotometer, and then the RNA samples were cryopreserved in a refrigerator at -80℃ for next use.

#### Detection of GATA-3, RORγt and FoxP3 mRNA levels

With total RNA of mononuclear cells as template and GAPDH as internal reference gene (forward primer: 5’CAAGGCTGTGGGCAAGGTCATC-3’, reverse primer: 5’-GTGTCGCTGTTGAAGTCAGAGGAG-3’), the reaction system was added according to the instructions of the FastKing one-step RT-PCR kit produced by Tiangen Biotech (Beijing) Co., Ltd. for reverse transcription (42℃ 30 min, 95℃ 3 min) and RT-PCR (94℃ 30 s, 65℃ 30s, 72℃ 30 s, 40 cycles, 72℃ 5 min). ABI 7500 real-time fluorescent quantitative PCR instrument was used for detecting the levels of GATA-3 mRNA (forward primer: 5’-CAGTTGGCCTAAGGTGGTT-3’, reverse primer: 5’-GCACGCTGGTAGCTCATACA-3’), RORγt mRNA (forward primer: 5’-GCTGTGATCTTGCCCAGAACC-3’, reverse primer: 5’-CTGCCCATCATTGCTGTTAATCC-3’), and FoxP3 mRNA (forward primer: 5’-CTGCCCCTAGTCATGGTGG-3’, reverse primer: 5’-CTGGAGGAGTGCCTGTAAGTG-3’). Replicates were made for each sample. After reaction, the melting curve was drawn to distinguish specific and non-specific amplification, and the relative expressions of target genes were calculated by the 2^–ΔΔ^^CT^ method.

#### Count of eosinophils and basophils in peripheral blood

Whole blood cell count was carried out using EDTA-K2 anticoagulant blood. The internal quality control of samples was conducted before detection. All detection items were tested by BC-7500CS full-automatic blood cell counter and its supporting reagents within 1 h after blood collection.

1.2.6 Detection of serum cytokines IL-4, IL-5, IL-17, IL-10 and IgE.

After collection by coagulation-promoting tube, blood was placed at room temperature for 30 min. After coagulation, blood was centrifuged at 3,500 r/min for 10 min (with a centrifugal radius of 10 cm), and the serum was divided into two parts. One part was used to detect the concentration of IgE by E601 fully automatic electrochemiluminescence immunoassay analyzer and its supporting reagents, with internal quality control before sample detection. The other part was used for detection of cytokines IL-4, IL-5, IL-17 and IL-10 using an ELISA kit according to the instructions. OD value was measured at 450 nm by a microplate reader, and cytokine concentrations were calculated according to the standard curve.

#### Main instruments and reagents

The main instruments and reagents used are as follows: BC-7500CS full-automatic blood cell counter (Mindray, China), E601 fully automated electrochemiluminescence immunoassay analyzer (Roche, USA), BD FACSCalibur flow cytometer (BD, USA), ABI 7500 real-time fluorescent quantitative PCR instrument (Thermo Fisher, USA), DW-86L388 -80℃ ultra-low temperature refrigerator (Haier, China), lysing solution, perm/wash buffer, stimulant and IL-4/IL-17 A/CD25/CD4/FoxP3 fluorescent antibody (BD, USA), RNA Easy Fast total RNA extraction kit and FastKing one-step RT-PCR kit (Tiangen Biotech Co., Ltd., Beijing), and human IL-4, IL-5, IL-10, IL-17 ELISA kit (NeoBioscience Technology Co., Ltd., Shenzhen).

### Statistical methods

The data were analyzed using SPSS 27.0. The counting data were expressed as rate, and their inter-group comparisons were conducted by the *χ*^2^ test. The measurement data conforming to the normal distribution were expressed as $\overline x + s$ and compared between groups using the *t* test or corrected *t*-test, and those of non-normal distribution were expressed as median and quartile [*M* (*P*_25_ ∼ *P*_75_)] and compared between groups with the nonparametric test. The correlation between the two groups was analyzed by linear regression. *P* < 0.05 was considered as statistically significant.

## Results

### Comparison of clinical data

In the AR group (*n* = 200), there were 101 males and 99 females, with an average age of 15.0 (10.0 ∼ 32.0) years. The HC group (*n* = 50) included 28 males and 22 females, with an average age of 17.5 (13.0 ∼ 30.3) years. Gender and age composition showed no statistically significant differences between the AR group and the HC group (*P* > 0.05), suggesting comparability. The levels of IgE, eosinophils and basophils in the peripheral blood of the AR group were higher than those of the HC group, with statistically significant differences (*P* < 0.05), as seen in Table 1.

**Table 1:** Comparison of clinical data between the two groups

Indicator	HC group (*n* = 50)	AR group (*n* = 200)	χ^2^/Z	*P*
Gender				
Male (*n*)	28(56.0%)	101(50.5%)	0.484	0.486
Female (*n*)	22(44.0%)	99(49.5%)		
Year (year)	17.5(13.0∼30.3)	15.0(10.0∼32.0)	-1.028	0.304
IgE (IU/ml)	18.985(10.505∼31.868)	199.650(74.825∼426.425)	-9.564	< 0.001
Eosinophils (10^9^/L)	0.125(0.060∼0.173)	0.430(0.283∼0.708)	-8.533	< 0.001
Basophils (10^9^/L)	0.025(0.018∼0.040)	0.050(0.040∼0.070)	-7.462	< 0.001

Note: AR, allergic rhinitis; HC, healthy control

### Comparison in relative expression of transcription genes

**Fig. 1:**
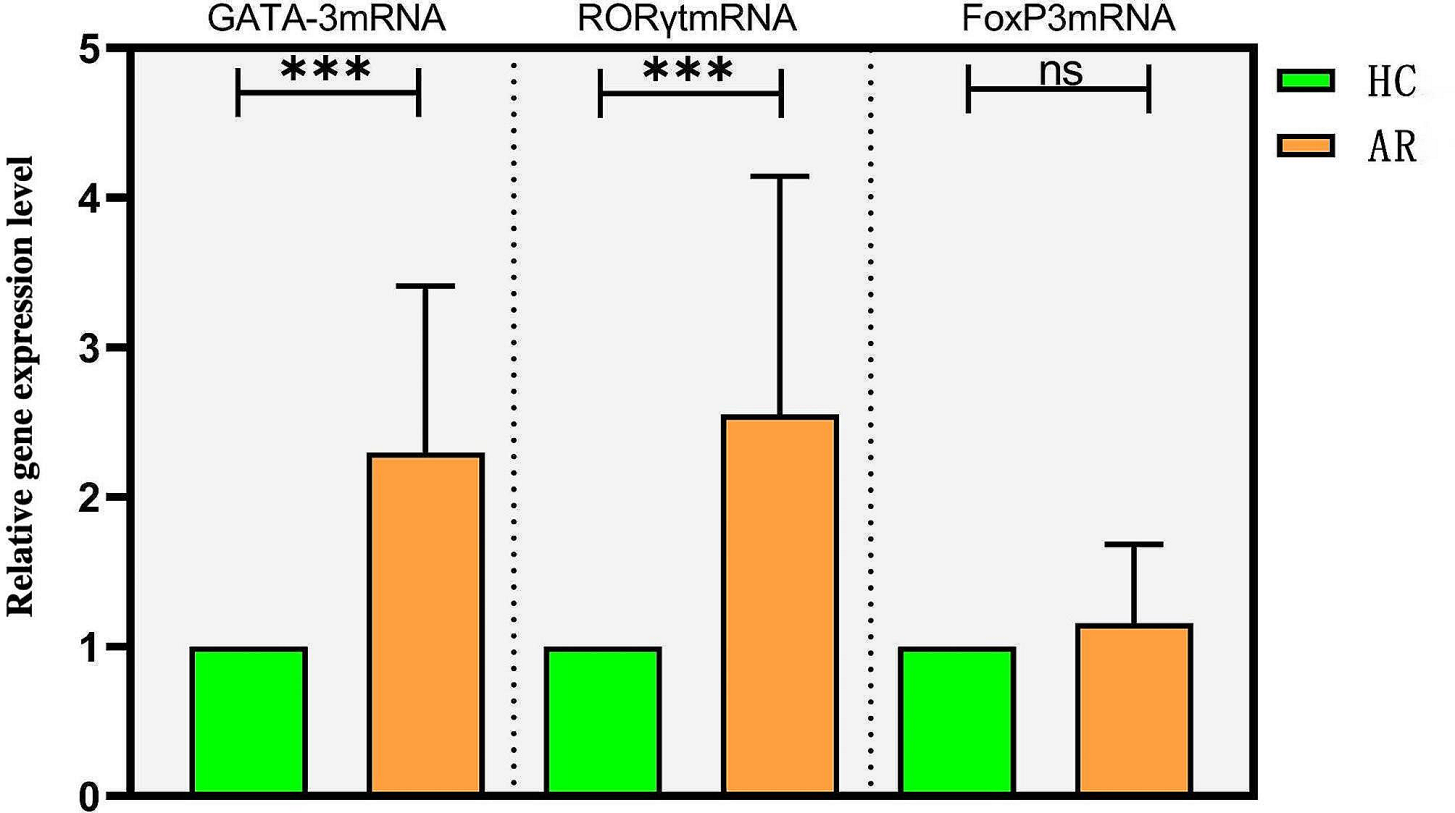
Comparison of relative mRNA expressions of transcription genes GATA-3, RORγt and FoxP3 between the two groups (Notes: ***: *P* < 0.001, ns, *P* > 0.05. AR, allergic rhinitis; HC, healthy control)

Compared with the HC group, the relative mRNA expressions of transcription genes GATA-3 and RORγt in peripheral blood mononuclear cells of AR patients increased significantly (2^−ΔΔCt^ > 2), with significant differences (*P* < 0.05). The relative mRNA expression of transcription gene Foxp3 increased, but the difference was not significant (*P* > 0.05), as shown in Table 2; Fig. 1.

**Table 2:** Comparison in relative expression of transcription genes between the two groups

Transcription gene	HC Gourp (*n* = 50)		AR Gourp (*n* = 200)		Z value	*P* value
	ΔCt	2 ^−ΔΔCt^	ΔCt	2 ^−ΔΔCt^		
GATA-3mRNA	0.830(-0.750∼2.618)	1	-0.370(-2.000∼-0.740)	2.300	-5.144	< 0.001
RORγtmRNA	1.170(0.195∼2.435)	1	-0.180(-3.258∼2.603)	2.549	-2.014	< 0.001
FoxP3mRNA	0.775(-0.745∼1.515)	1	0.570(-1.540∼2.090)	1.153	-0.828	0.407

Note: AR, allergic rhinitis; HC, healthy control

### Comparison of immune cell proportion in peripheral blood

**Fig. 2:**
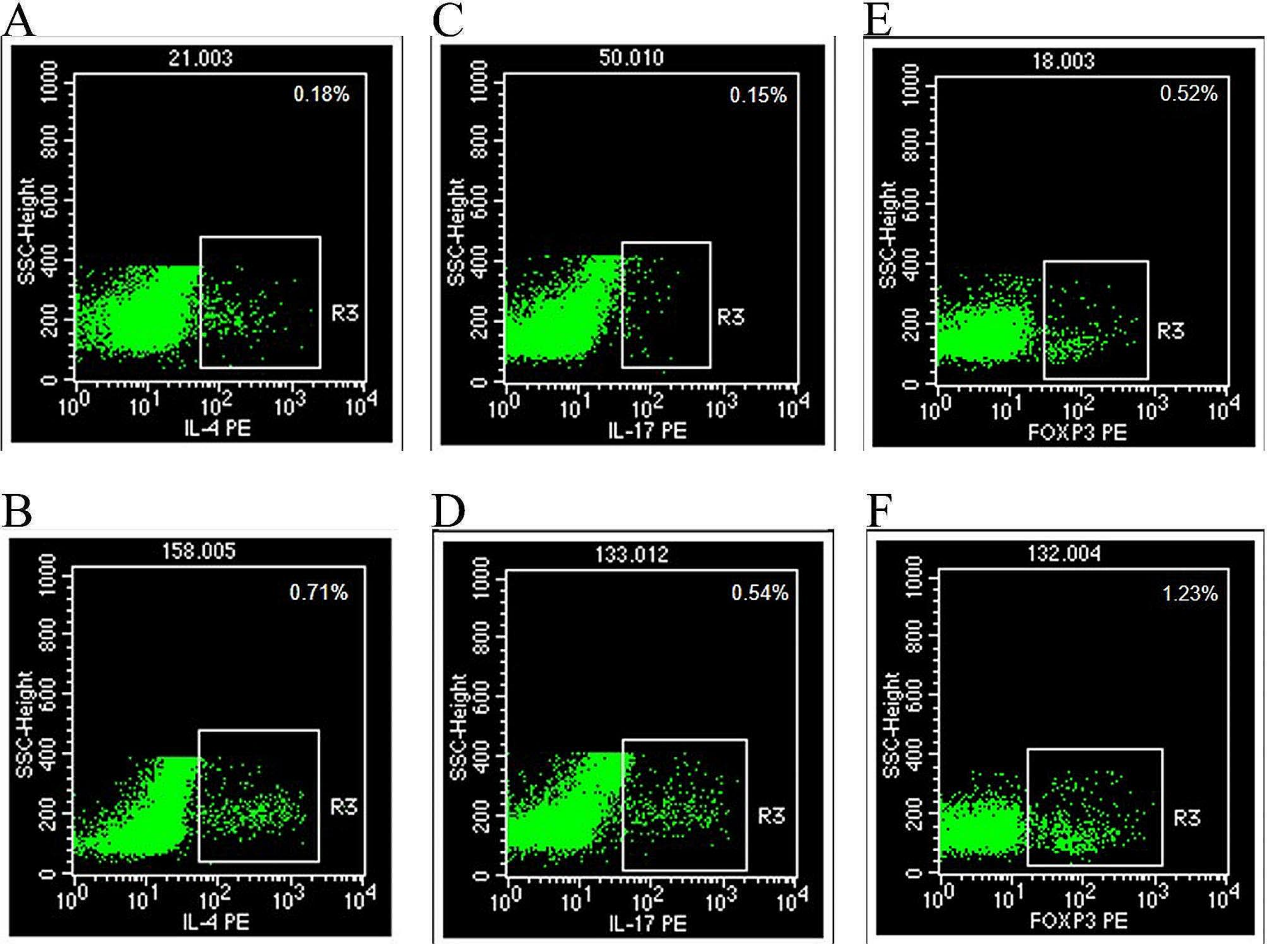
Flow cytometry for detection of Th2, Th17 and Treg cells. **A**: Detection map of Th2 cells in the HC group; **B**: Detection map of Th2 cells in the AR group; **C**: Detection map of Th17 cells in the HC group; **D**: Detection map of Th17 cells in the AR group; **E**: Detection map of Treg cells in the HC group; **F**: Detection map of Treg cells in the AR group. Note: AR, allergic rhinitis; HC, healthy control

**Fig. 3:**
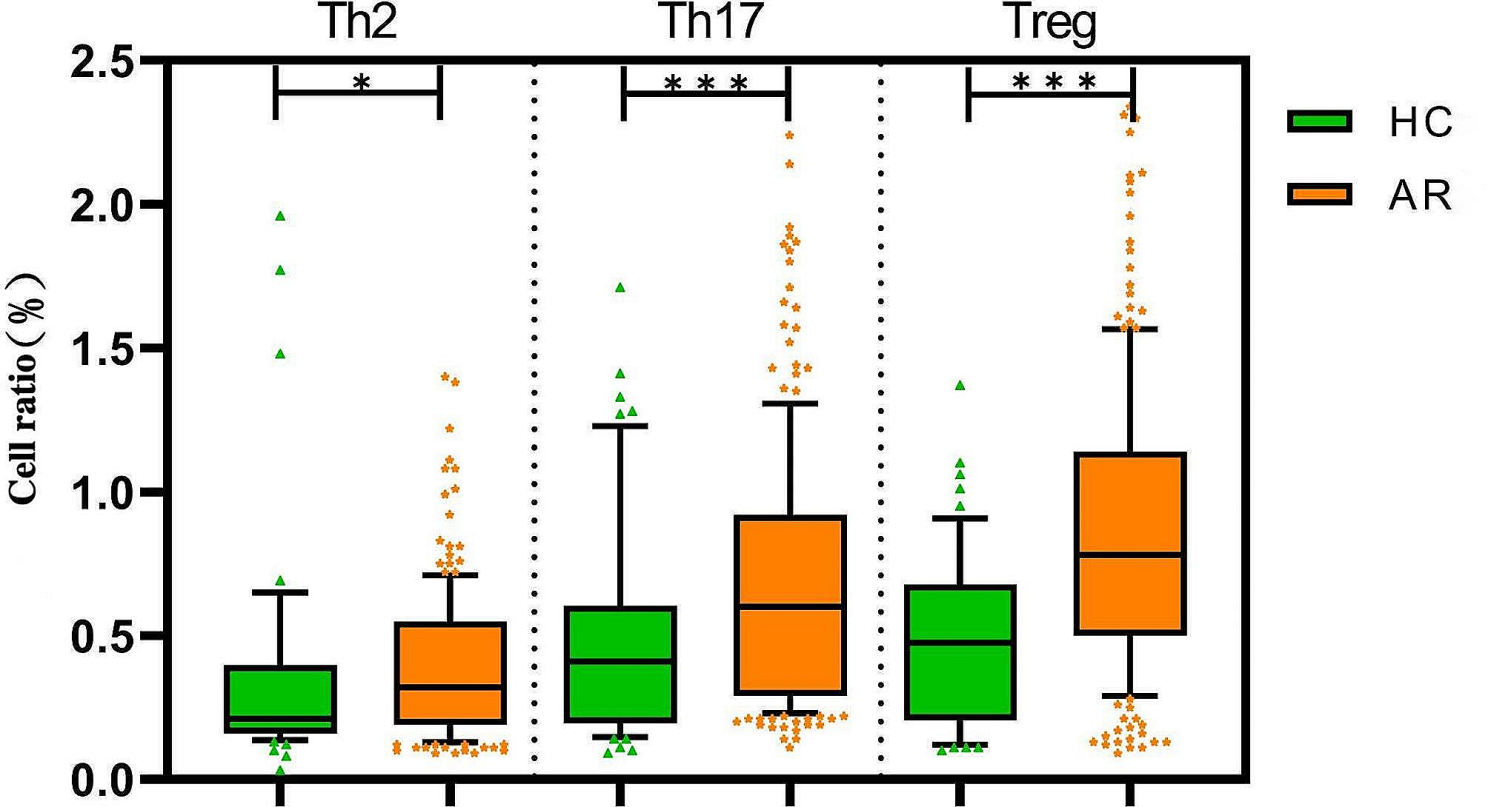
Box plot for comparison of Th2, Th17 and Treg cells between the two groups (Notes: *: *P* < 0.05, ***: *P* < 0.001. AR, allergic rhinitis; HC, healthy control)

The proportions of Th2, Th17 and Treg cells in the peripheral blood of AR patients were higher than those of the HC group, with statistically significant differences (*P* < 0.05), as presented in Table 3; Figs. 2 and 3.

**Table 3:** Comparison of immune cells between the two groups (%)

Immune cells	HC group (*n* = 50)	AR group (*n* = 200)	Z	*P*
Th2	0.210(0.160∼0.398)	0.320(0.190∼0.550)	-2.424	0.015
Th17	0.410(0.195∼0.605)	0.600(0.290∼0.920)	-3.418	< 0.001
Treg	0.475(0.205∼0.678)	0.780(0.500∼1.140)	-5.229	< 0.001

Note: AR, allergic rhinitis; HC, healthy control

### Comparison of serum cytokine levels

**Fig. 4:**
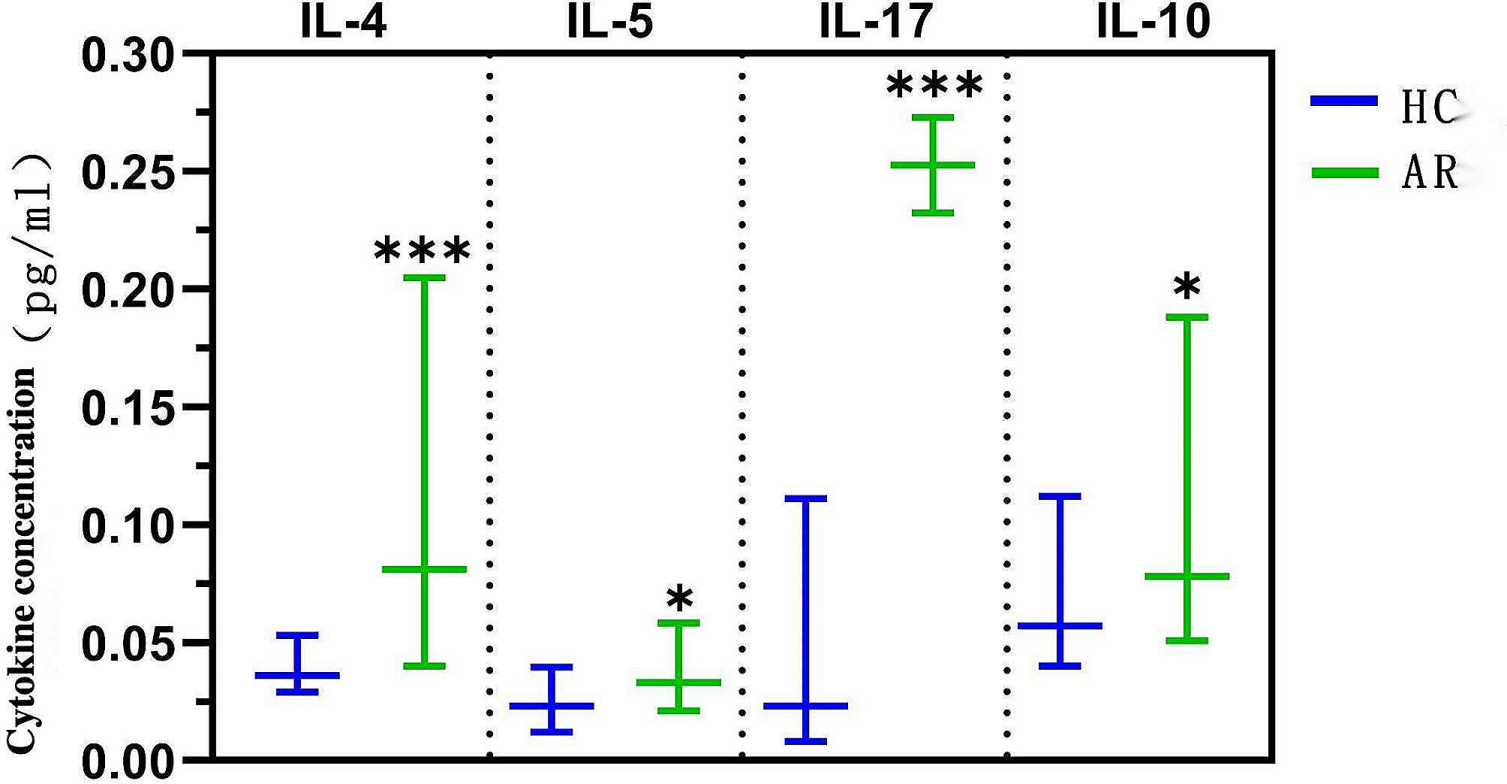
Comparison of cytokine IL-4, IL-5, IL-17 and IL-10 concentrations between the two groups (Notes: *: *P* < 0.05, ***: *P* < 0.001. AR, allergic rhinitis; HC, healthy control)

The concentrations of IL-4, IL-5, IL-17 and IL-10 in the serum of AR patients were higher than those of the HC group, with statistically significant differences (*P* < 0.05), as exhibited in Table 4; Fig. 4.

**Table 4:** Comparison of cytokine concentrations between the two groups (pg/ml)

Cytokine	HC group(*n* = 50)	AR group(*n* = 200)	Z value	*P* value
IL-4	0.036(0.029∼0.053)	0.081(0.040∼0.205)	-4.288	< 0.001
IL-5	0.023(0.012∼0.040)	0.033(0.021∼0.059)	-2.350	0.019
IL-17	0.023(0.008∼0.111)	0.161(0.060∼0.324)	-5.493	< 0.001
IL-10	0.057(0.040∼0.112)	0.078(0.051∼0.188)	-2.252	0.024

Note: AR, allergic rhinitis; HC, healthy control

### Correlations of cytokines IL-4 and IL-17 with IgE

**Fig. 5:**
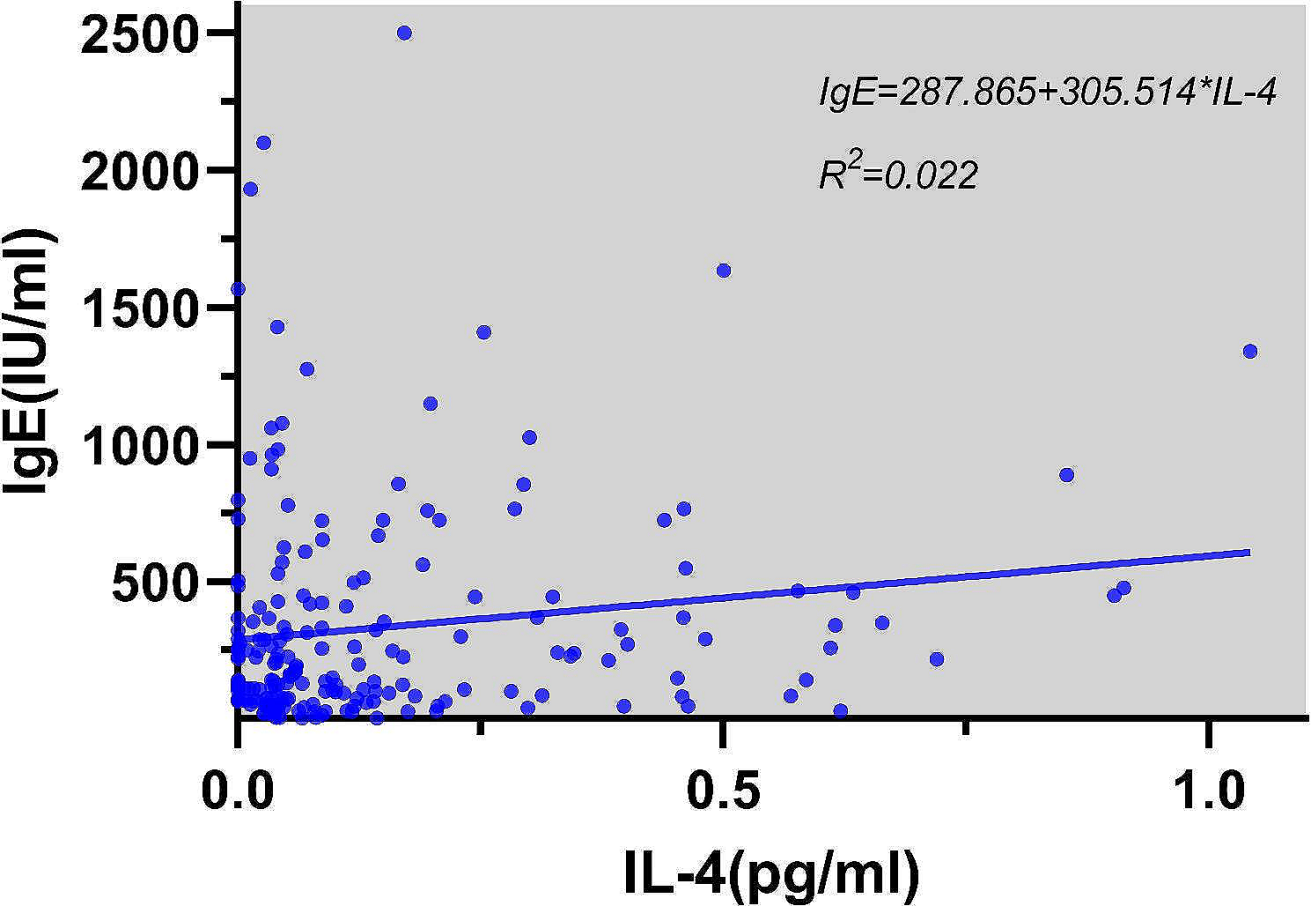
Linear regression for the effect of IL-4 on IgE

**Fig. 6:**
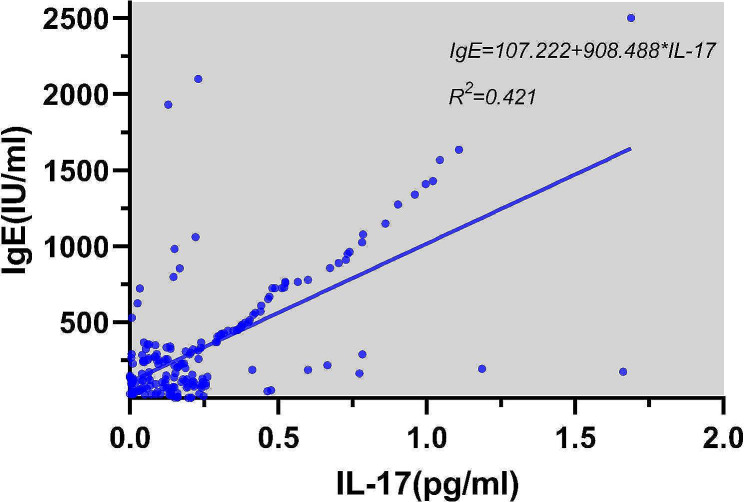
Linear regression for the effect of IL-17 on IgE

Through linear regression analysis, the concentration of IL-4 in the serum of AR patients had a weakly positive correlation with IgE content, and the increase in IL-4 concentration was one of the factors associated with the increase in serum IgE content of AR patients (*R*^2^ = 0.022, *P* = 0.035). There was a strong positive correlation between IL-17 concentration and IgE content. IL-17 was one of the main factors associated with the increase in serum IgE content of AR patients (*R*^2^ = 0.421, *P* < 0.001). (Figures 5 and 6). Statistical analysis showed no significant correlations of Th2, Th17 and Treg cells with IgE and cytokines.

## Discussion

In the present study, the expressions of Th2, Th17 and Treg-related transcription factors and cytokines in the peripheral blood from 200 AR patients and 50 healthy individuals were detected. The results showed that compared with the HC group, the mRNA levels of transcription genes GATA-3 and RORγt in peripheral blood mononuclear cells of AR patients, percentages of immune cells Th2, Th17 and Treg in the peripheral blood, eosinophil and basophil levels, as well as cytokines IL-4, IL-5, IL-17 and IL-10 and IgE in the serum significantly increased. In addition, IL-4 and IL-17 were positively correlated with total IgE level. These findings provide important implications for the pathogenesis of AR.

AR is IgE-induced type I hypersensitivity, which is related to the body contact with allergens, abnormal activation of immune cells and immune imbalance. In this study, the levels of eosinophils, basophils and serum IgE in the peripheral whole blood of AR patients were significantly higher than those in the HC group. After sensitization with Artemisia argyi, the number of bioactive mediators released by eosinophils and basophils in AR patients increased, which triggered a strong immunological effect.

Under immune balance of the body, Th1 and Th2 cells mutually antagonize and regulate each other. When the body is stimulated by allergens, Th1/Th2 imbalance leads to abnormal immune response, which has been the main immunological basis and pathogenesis of AR [8]. Treg and Th17 cells are newly discovered CD4^+^ T cell subsets. Th17 cells can induce inflammatory tissue injuries by recruiting inflammatory cells, and Treg cells play an important role in maintaining immune homeostasis [9]. Treg and Th17 cells also jointly regulate the immune tolerance and stability of the body, which is closely related to the occurrence and development of AR [10]. In this study, the levels of Th2, Th17 and Treg cells in the peripheral blood of the AR and HC groups were detected by flow cytometry, revealing that the proportions of Th2, Th17 and Treg cells in the peripheral blood of AR patients were significantly higher than those of healthy individuals, indicating high proliferation and activation of immune cells Th2, Th17 and Treg in AR patients, which is involved in the pathogenesis of AR.

Immune cells exert immune effects mainly through cytokines they secrete. Th2 cell differentiation can produce a large number of cytokines, including IL-4, IL-5, IL-9 and IL-13, which induce B cells to produce antigen-specific IgE, promoting the development of inflammatory response and the migration of eosinophils to local tissues [11]. IL-17 is mainly produced by Th17 cells and mediates inflammatory response and autoimmune diseases [12]. Human Treg cells are a group of T cells with negative immune regulation, which can secrete IL-10 and TGF-β that play an important role in maintaining the balance between immune response and immune tolerance [13]. In our study, the concentrations of cytokines IL-4, IL-5, IL-17 and IL-10 in the serum of AR patients were significantly higher than those of the HC group, which is mainly caused by increased proportions of Th2, Th17 and Treg cells in AR patients, and correspondingly increased cytokines secreted into the blood. These cytokines participate in the occurrence and development of AR by exerting an immune effect.

Through linear regression analysis, it was also found that serum IL-4 concentration in AR patients was weakly positively correlated with IgE content, and the increase in IL-4 concentration was one of the causes for the increase of serum IgE content in AR patients. Additionally, a strong positive correlation was found between IL-17 concentration and IgE content, and IL-17 was one of the main reasons for the increase in serum IgE content of AR patients, indicating that IL-4 and IL-17 are involved in the regulation of IgE production during the pathogenesis of AR, thereby inducing the occurrence of allergic reactions. Inhibiting the production of IL-4 and IL-17 and reducing the concentrations of IL-4 and IL-17 in vivo may become new methods and targets for the treatment of AR.

These findings are consistent with previous research [14] and provide important implications for the pathogenesis of AR. The increase in IL-4 concentration was associated with the increase in serum IgE content in AR patients in our study, which aligns with the existing understanding that IL-4 induces B cells to produce antigen-specific IgE [15]. Additionally, a strong positive correlation was observed between IL-17 concentration and IgE content, suggesting that IL-17 may be associated with increased serum IgE levels in AR patients, although further research is needed to establish causality.

GATA-3 can promote Th2 response through three different mechanisms: induction of Th2-related cytokine production, selective growth of Th2 cells, and inhibition of Th1 cell-specific factors [16]. Transcription factor RORγt is closely related to the production of Th17 cells. RORyt with ligand binding sites is a necessary condition for the induction of IL-17. It has been shown that RORγt is deficient in T cells of mice, and the incidence of autoimmune diseases and Th17 cell infiltration in relevant tissues are reduced [17, 18]. Transcription factor FoxP3 plays an important role in regulating the differentiation, development and function of Treg cells [19]. FoxP3 deficiency leads to the loss of CD4 + CD25 + Treg cells. Through the research on scurfy mice and human patients with immune disorders, multiple endocrine failure, intestinal lesions and X chromosome-associated syndrome, it has been found that transcription factor FoxP3 plays an important role in maintaining self-tolerance [18, 20]. In this study, the mRNA levels of transcription genes GATA-3, RORγt and FoxP3 in peripheral blood mononuclear cells of the AR and HC groups were detected using real-time fluorescent quantitative PCR, which revealed that the relative expressions of GATA-3 mRNA, RORγt mRNA and FoxP3 mRNA in the peripheral blood of AR patients were higher than those of healthy individuals. It is indicated that the occurrence of AR is related to the regulation of transcription genes GATA-3 mRNA, RORγt mRNA and FoxP3 mRNA, the proliferation and differentiation of immune cells Th2, Th17 and Treg, and the biological effects of cytokines IL-4, IL-5, IL-17 and IL-10 on target tissues. AR and its symptom aggravation may be caused through the gene regulation-cell proliferation-cytokine secretion axis.

In our study, GATA-3 mRNA and RORγt mRNA levels, the proportions of Th2 and Th17 cells, and the concentrations of cytokines IL-4, IL-5 and IL-17 all increased in AR patients compared with those in HCs, which is consistent with many existing research results [21–24]. The difference is that the mRNA level of transcription gene FoxP3, immune cells Treg and cytokine IL-10 in AR patients were also higher than those in HCs in this study. The results of Li Zhi [25], Tian Jingqi [26], and Boonpiyathad T [27] show that immune cells Treg and cytokine IL-10 in the peripheral blood of AR patients are reduced. This may be related to the different sensitization effects of Artemisia desertorum pollen allergens in Ordos and other allergens, the complex immune regulation mechanism involved in AR, the strong immunosuppressive effect of the body and the high prevalence rate. Therefore, further grouping study on AR patients sensitized by other allergens in this region is needed for confirmation.

This study has some limitations that should be addressed in future research. Firstly, the sample selection was limited to specific AR patients sensitized to Artemisia allergens and a control group, which may not be representative of AR patients caused by other allergens. Additionally, the study focused on a specific population sensitized to Artemisia allergens in the Ordos region, which may limit the generalizability of the findings to other allergens or geographic regions. Future studies should include diverse allergen-specific groups and a broader range of allergens and diverse patient populations from different geographic areas to enhance generalizability.Secondly, the smaller number of healthy controls included in our study may have limited its statistical power, which could be addressed by recruiting a larger control group in future investigations.Moreover, the cross-sectional design of our study does not allow for causal inferences between the observed immunological changes and AR symptomatology. Further longitudinal or interventional studies are needed to establish causal relationships and explore the potential mechanisms underlying the observed associations.Additionally, our study did not account for potential confounding factors such as comorbidities, medication history, or environmental exposures, which could influence immune parameters and cytokine levels. Controlling for these factors in future studies would enhance the internal validity of the results and provide a more comprehensive understanding of the immunological changes associated with AR.

Future studies could investigate the efficacy of agents that selectively inhibit the production or activity of IL-4 and IL-17, or target the transcriptional regulation of GATA-3 and RORγt, thereby modulating the differentiation and function of Th2 and Th17 cells, respectively. Additionally, strategies aimed at restoring the balance between effector T cells (Th2, Th17) and regulatory T cells (Treg) could be explored, potentially through the use of agents that enhance Treg cell function or promote their differentiation via modulation of FoxP3 expression. Furthermore, the gene regulation-cell proliferation-cytokine secretion axis, identified as a potential pathway involved in AR pathogenesis, could be targeted through novel therapeutic interventions. Overall, the immunological factors identified in this study provide a foundation for future research to develop targeted, personalized treatment approaches for AR.

## Conclusion

In conclusion, immune cells and cytokine secretion in the peripheral blood of AR patients are abnormal. The results suggest that Th2, Th17 and Treg-specific transcription factors and the relevant cells and cytokines are associated with the occurrence and development of AR in our study. The gene regulation-cell proliferation-cytokine secretion axis may play an important role in the pathogenesis of AR. The results will improve the in-depth understanding of AR mechanism and provide new ideas for the treatment of AR.

## Data Availability

All data generated or analyzed during this study are included in this published article.
